# Role of immunohistochemistry in differentiating between reactive and malignant lymphoid hyperplasia - A cross-sectional study

**DOI:** 10.6026/973206300213470

**Published:** 2025-10-31

**Authors:** Sakshi Chourasia, Seema Badkur, Ajit Kumar, Mitesh Shah

**Affiliations:** 1 Department of Pathology, Government Medical College, Satna, Madhya Pradesh, India; 2 Department of Community Medicine, Gandhi Medical College, Bhopal, Madhya Pradesh, India; 3 Department of Radiation Oncology, SSMC and Sanjay Gandhi Memorial Hospital, Rewa, Madhya Pradesh, India; 4 Department of Pathology, Bundelkhand Medical College, Sagar, Madhya Pradesh, India

**Keywords:** Immunohistochemistry, reactive lymphoid hyperplasia, malignant lymphoma, diagnostic differentiation, cross-sectional study

## Abstract

Differentiating reactive lymphoid hyperplasia from malignant lymphoma poses a diagnostic challenge due to overlapping clinical and histopathological features. Therefore, it is of interest to evaluate the utility of immunohistochemistry (IHC) in distinguishing benign reactive changes from malignant lymphoid proliferations. Lymph node biopsies from patients presenting with persistent lymphadenopathy were assessed histologically and subjected to a panel of IHC markers including CD3, CD20, CD10, BCL2 and Ki-67. The analysis revealed that IHC not only confirmed lineage specificity but also helped to identify aberrant antigen expression and proliferative indices characteristic of malignancy. Thus, we show the pivotal role of IHC in improving diagnostic accuracy, reducing misclassification and guiding appropriate therapeutic decisions in patients with lymphoid hyperplasia.

## Background:

Lymphoid hyperplasia encompasses a heterogeneous group of disorders characterized by the proliferation of lymphoid tissue, which may be reactive or malignant in nature. Reactive lymphoid hyperplasia represents a benign, polyclonal immune response triggered by antigenic stimulation from infections, autoimmune conditions, or chronic inflammatory states [1]. In contrast, malignant lymphoid hyperplasia, most commonly in the form of non-Hodgkin and Hodgkin lymphomas, arises from monoclonal proliferation of lymphocytes due to genetic and molecular alterations that disrupt normal growth and differentiation pathways [2]. The clinical presentation of both conditions often overlaps, typically manifesting as lymphadenopathy, systemic constitutional symptoms and in some cases, extranodal involvement [3]. This clinical similarity makes accurate differentiation a critical diagnostic challenge. Histopathological examination has long been the primary modality for evaluating lymphoid lesions. Reactive hyperplasia demonstrates preserved architecture with heterogeneous lymphocyte populations and active germinal centers, while malignant lymphomas often exhibit effaced nodal architecture, cytological atypia and monoclonality. However, significant morphological overlap exists in early or borderline cases, where atypical reactive processes may mimic malignancy and low-grade lymphomas may resemble benign hyperplasia [4]. Consequently, reliance on morphology alone is insufficient for conclusive diagnosis, often leading to misclassification that has profound implications for patient management. Immunohistochemistry (IHC) has emerged as an indispensable adjunct in the diagnostic algorithm for lymphoid lesions. By enabling the detection of specific antigenic markers, IHC provides insights into lineage determination, clonality and cellular proliferation [5]. T-cell markers such as CD3, B-cell markers like CD20 and PAX5 and germinal center markers including CD10 and BCL6, allow for lineage assignment and distinction between reactive and neoplastic processes. Aberrant expression of markers such as BCL2 in germinal centers, or the loss of expected markers, can serve as powerful indicators of malignancy. Additionally, proliferative indices assessed by Ki-67 staining offer quantifiable measures of cellular turnover, with higher values strongly suggestive of malignant transformation [6]. Beyond basic lineage identification, IHC also plays a role in subtyping lymphomas, which has direct therapeutic and prognostic implications. Distinguishing between reactive follicular hyperplasia and follicular lymphoma, or between reactive paracortical hyperplasia and peripheral T-cell lymphoma, is often impossible without IHC-based assessment. Moreover, panels of markers, rather than single antigen evaluation, have been shown to provide higher diagnostic accuracy by accounting for the heterogeneity within lymphoid neoplasms [7]. The refinement of IHC techniques and the introduction of more specific markers have further enhanced its role as a gold standard tool in lymphoid pathology. Accurate differentiation between reactive and malignant lymphoid hyperplasia is essential not only for avoiding unnecessary aggressive therapy in benign conditions but also for preventing delays in the treatment of true malignancies [8]. Misdiagnosis can lead to either overtreatment with cytotoxic regimens, causing avoidable morbidity, or undertreatment of malignant disease, resulting in disease progression and poor prognosis. Incorporating IHC into standard diagnostic workflows thus ensures greater diagnostic precision and contributes to evidence-based patient care [9]. Therefore, it is of interest to describe the role of immunohistochemistry in differentiating between reactive and malignant lymphoid hyperplasia through a cross-sectional study approach.

## Materials and Methods:

This cross-sectional study was conducted in the Department of Pathology at a tertiary care teaching hospital over a two-year period. All patients presenting with clinically significant lymphadenopathy who underwent excisional lymph node biopsy were considered for inclusion. Cases with inadequate tissue, prior history of chemotherapy or radiotherapy, or extensive necrosis were excluded. The study population included individuals of all age groups and both sexes and informed consent was obtained from each participant. Excisional biopsy specimens were fixed in 10% buffered formalin, processed using standard histopathological techniques and stained with hematoxylin and eosin for evaluation. Histological examination focused on preservation of nodal architecture, presence of germinal centers, degree of cytological atypia and mitotic activity, thereby allowing categorization into reactive lymphoid hyperplasia and suspected malignant lesions. For confirmatory assessment, immunohistochemistry was performed on representative tissue sections cut at 4 μm thickness and mounted on poly-L-lysine-coated slides. Antigen retrieval was carried out in citrate buffer (pH 6.0) using a pressure cooker method, followed by inhibition of endogenous peroxidase activity. Primary antibodies against CD20, PAX5, CD3, CD5, CD10, BCL6, BCL2 and Ki-67 were applied, with the streptavidin-biotin technique and diaminobenzidine chromogen used for visualization. Positive and negative controls were included for all markers. Interpretation of immunohistochemical staining was performed independently by two experienced pathologists to minimize interobserver variability. Cases exhibiting diffuse strong expression of lineage markers, aberrant immunophenotypes and high proliferative indices were considered malignant, while those demonstrating polyclonal staining and preserved nodal heterogeneity were classified as reactive hyperplasia. Clinical details, including age, sex, duration of illness, systemic symptoms and relevant medical history, were retrieved from patient records and correlated with the histopathological and immunohistochemical findings. The collected data were analyzed descriptively to assess the diagnostic utility of immunohistochemistry in differentiating between reactive and malignant lymphoid hyperplasia.

## Results:

The present cross-sectional study analyzed excisional lymph node biopsies to evaluate the role of immunohistochemistry (IHC) in distinguishing reactive lymphoid hyperplasia from malignant lymphoid lesions. A total of 120 cases were included, encompassing a broad age range and both sexes. Histopathological examination provided preliminary differentiation, but certain cases with overlapping features necessitated the use of IHC markers for definitive categorization. The panel of antibodies including CD20, PAX5, CD3, CD5, CD10, BCL6, BCL2 and Ki-67 significantly enhanced diagnostic accuracy by highlighting clonal proliferation patterns and proliferative indices. The combined approach of histopathology and immunohistochemistry allowed clear separation of benign from malignant lesions in most cases, minimizing diagnostic ambiguity. The results underscore the pivotal role of IHC in resolving challenging cases, particularly those with atypical histological features.

Table 1 (see PDF) shows the distribution of patients according to age, sex and clinical presentation. It highlights that younger patients frequently presented with reactive hyperplasia, whereas older individuals had higher rates of malignant pathology. Table 2 (see PDF) demonstrates the association between systemic symptoms and diagnosis, indicating that B-symptoms were predominantly seen in malignant lymphomas, whereas reactive hyperplasia often presented with localized lymphadenopathy only. Table 3 (see PDF) highlights the morphological differences observed between reactive and malignant cases, showing that architectural effacement and cytological atypia were strongly indicative of malignancy. Table 4 (see PDF) shows the expression of lineage-specific markers, demonstrating the diagnostic separation between polyclonal reactive proliferations and monoclonal malignant proliferations. Table 5 (see PDF) demonstrates the improvement in diagnostic precision achieved with IHC, particularly in morphologically ambiguous cases. Table 6 (see PDF) classifies malignant lymphomas identified in the study, showing that diffuse large B-cell lymphoma was the most frequent subtype. Table 7 (see PDF) demonstrates marker expression patterns that helped subtype malignant lymphomas, confirming their diagnostic utility. Table 8 (see PDF) shows that malignant lymphomas had a significantly higher Ki-67 index compared to reactive hyperplasia, supporting its role in differentiating aggressive proliferations. Table 9 (see PDF) highlights that IHC was particularly useful in resolving discrepancies, with high concordance in straightforward cases but diagnostic revisions in 22% of ambiguous cases. Table 10 (see PDF) summarizes the final diagnostic categorization of all cases, illustrating the impact of IHC in achieving definitive classification.

## Discussion:

The present study underscores the critical role of immunohistochemistry (IHC) in differentiating between reactive lymphoid hyperplasia and malignant lymphoid proliferations. While routine histopathology remains the cornerstone of diagnosis in lymphoid lesions, the inherent overlap in architectural and cytological features between benign and malignant processes frequently poses diagnostic challenges [10]. IHC bridges this diagnostic gap by providing a molecular and phenotypic signature of lymphoid cells, thereby enabling accurate classification and subtyping. The demographic distribution in the study reflected the clinical observation that lymphoid hyperplasia commonly affects younger adults, whereas malignant lymphomas are relatively more frequent in older individuals [11]. This aligns with global data on lymphoid neoplasms, which demonstrate age-dependent variation in incidence and presentation. The male predominance observed has also been noted in multiple epidemiological reports, suggesting possible hormonal and immunological influences in disease susceptibility [12]. Histopathology alone, although highly informative, can be confounded by reactive follicular hyperplasia mimicking follicular lymphoma or by atypical hyperplastic changes raising suspicion of malignancy. In such cases, IHC proved invaluable in validating the diagnosis [13]. For instance, the expression of CD3 and CD20 allowed for lineage confirmation, while markers such as BCL2, BCL6 and CD10 were indispensable for distinguishing follicular lymphoma from reactive hyperplasia. Similarly, the Ki-67 proliferation index served as a reliable adjunct in differentiating indolent reactive conditions from high-grade malignant lymphomas, given the significantly elevated proliferative activity in the latter [14]. The diagnostic accuracy of IHC was further highlighted by its ability to revise the initial histopathological impression in nearly one-fourth of cases. This demonstrates that sole reliance on morphology can lead to under- or over-diagnosis, with potential consequences on patient management [15]. Integrating IHC minimized these risks, leading to a more robust and reproducible diagnosis. Moreover, IHC provided insights into sub classification of lymphomas, particularly differentiating diffuse large B-cell lymphoma (DLBCL), follicular lymphoma and Hodgkin's lymphoma, each of which necessitates distinct therapeutic strategies [16]. From a clinical perspective, the findings have direct implications in guiding therapy [17].

Patients with malignant lymphomas identified through IHC could be referred for early chemotherapy or targeted therapies, while those confirmed with reactive hyperplasia were spared from unnecessary aggressive treatment [18]. This diagnostic precision not only optimizes patient outcomes but also reduces the healthcare burden by preventing overtreatment. The study also demonstrates the global relevance of a standardized IHC panel in lymphoid pathology [19]. Although the markers applied may vary slightly across institutions depending on availability and case complexity, the fundamental role of lineage markers (CD3, CD20), germinal center markers (CD10, BCL6) and proliferative indices (Ki-67) is universally acknowledged. Expanding marker panels may further refine the diagnostic landscape, especially with the advent of molecular pathology and integration of next-generation sequencing [20]. Limitations of the present study include its cross-sectional design, which precludes longitudinal assessment of disease outcomes. Furthermore, variability in marker interpretation across laboratories may influence reproducibility. Despite these limitations, the evidence strongly supports the indispensable role of IHC as a complementary tool to histopathology in the accurate differentiation of reactive and malignant lymphoid lesions. The study reinforces that while histopathology remains the backbone of diagnostic hematopathology; immunohistochemistry significantly enhances diagnostic accuracy, facilitates lymphoma subtyping and ensures appropriate patient management. The integration of both approaches should be considered the diagnostic gold standard in evaluating lymphoid hyperplasia.

## Conclusion:

Immunohistochemistry plays a pivotal role in enhancing the diagnostic accuracy of lymphoid lesions by reliably distinguishing reactive hyperplasia from malignant lymphoma. Its integration with conventional histopathology ensures precise classification, facilitates appropriate therapeutic decision-making and ultimately contributes to improved patient outcomes.
